# Tackling the burden of postsurgical complications in the USA: would perioperative goal-directed therapy help?

**DOI:** 10.1186/cc13313

**Published:** 2014-03-17

**Authors:** G Manecke, A Asemota, F Michard

**Affiliations:** 1 UCSD Medical Center, San Diego, CA, USA

## Introduction

Pay-for-performance programs and economic constraints call for solutions improving the quality of healthcare without increasing costs. Many studies have shown decreased morbidity in major surgery when perioperative goal-directed therapy (PGDT) is used. We assessed the clinical and economic burden of postsurgical complications in the University HealthSystem Consortium (UHC) in order to predict potential savings with PGDT.

## Methods

Data from adults who had 10 major surgical procedures in 2011 were screened in the UHC database. Thirteen postsurgical complications were tabulated. In-hospital mortality, hospital length of stay and costs from patients with and without complications were compared. The risk ratios reported by the most recent meta-analysis were used to estimate the potential reduction in postsurgical morbidity with PGDT. Potential cost savings were calculated from the actual and anticipated morbidity rates.

## Results

A total of 75,140 patients met the search criteria. In total, 8,421 patients developed one or more postsurgical complications (morbidity rate 11.2%). In-hospital mortality was 12.42% and 1.39% (*P *< 0.001), mean hospital length of stay was 20.48 ± 20.09 days and 8.5 ± 7.11 days (*P *< 0.001), and mean direct cost was $47,284 ± 49,170 and $17,408 ± 15,612 (*P *< 0.001) in patients with and without complications, respectively. With PGDT, morbidity rate was projected to decrease to 6.5 to 9.0%, yielding gross costs savings of $50 million to 151 million for the study population or $670 to $1,406 per patient.

## Conclusion

Postsurgical complications have a dramatic impact on mortality, hospital length of stay and costs. Potential cost savings resulting from PGDT are substantial. PGDT may be recommended to improve quality of care and decrease costs.

